# Comparisons of serum non-transferrin-bound iron levels and fetal cardiac function between fetuses affected with hemoglobin Bart’s disease and normal fetuses

**DOI:** 10.3389/fmed.2022.1015306

**Published:** 2023-01-03

**Authors:** Phudit Jatavan, Rattanaporn Sekararithi, Thidarat Jaiwongkam, Sirinart Kumfu, Nipon Chattipakorn, Theera Tongsong

**Affiliations:** ^1^Department of Obstetrics and Gynecology, Faculty of Medicine, Chiang Mai University, Chiang Mai, Thailand; ^2^Cardiac Electrophysiology Research and Training Center (CERT), Faculty of Medicine, Chiang Mai University, Chiang Mai, Thailand

**Keywords:** non-transferrin-bound iron, LPI, fetus, cardiac function, Hb Bart’s disease

## Abstract

**Objective:**

To compare the levels of Non-transferrin bound iron (NTBI) in fetuses with anemia, using Hb Bart’s disease as a study model, and those in unaffected fetuses and to determine the association between fetal cardiac function and the levels of NTBI.

**Patients and methods:**

A prospective study was conducted on pregnancies at risk of fetal Hb Bart’s disease. All fetuses underwent standard ultrasound examination at 18–22 weeks of gestation for fetal biometry, anomaly screening and fetal cardiac function. After that, 2 ml of fetal blood was taken by cordocentesis to measure NTBI by Labile Plasma Iron (LPI), serum iron, hemoglobin and hematocrit. The NTBI levels of both groups were compared and the correlation between NTBI and fetal cardiac function was determined.

**Results:**

A total of 50 fetuses, including 20 fetuses with Hb Bart’s disease and 30 unaffected fetuses were recruited. There was a significant increase in the level of serum iron in the affected group (median: 22.7 vs. 9.7; *p*-value: 0.013) and also a significant increase in NTBI when compared with those of the unaffected fetuses (median 0.11 vs. 0.07; *p*-value: 0.046). In comparisons of fetal cardiac function, myocardial performance (Tei) index of both sides was significantly increased in the affected group (left Tei: *p* = 0.001, Right Tei: *p* = 0.008). Also, isovolumetric contraction time (ICT) was also significantly prolonged (left ICT: *p* = 0.00, right ICT: *p* = 0.000). Fetal LPI levels were significantly correlated inversely with fetal hemoglobin levels (*p* = 0.030) but not significantly correlated with the fetal serum iron levels (*p* = 0.138). Fetal LPI levels were also significantly correlated positively with myocardial performance index (Tei) of both sides (right Tei: *R* = 0.000, left Tei: *R* = 0.000) and right ICT (*R* = 0.013), but not significantly correlated with left ICT (*R* = 0.554).

**Conclusion:**

Anemia caused by fetal Hb Bart’s disease in pre-hydropic stage is significantly associated with fetal cardiac dysfunction and increased fetal serum NTBI levels which are significantly correlated with worsening cardiac dysfunction. Nevertheless, based on the limitations of the present study, further studies including long-term data are required to support a role of fetal anemia as well as increased fetal serum NTBI levels in development of subsequent heart failure or cardiac compromise among the survivors, possibly predisposing to cardiovascular disease in adult life.

## Introduction

In our clinical practice, in a geographical area of high prevalence of thalassemia, we have noted that patients with alpha-thalassemia intermedia like Hb H disease or Hb H-Constant Spring (CS) disease seem to have higher prevalence of cardiovascular diseases or metabolic syndrome than that in normal population or even in beta-thalassemia patients. Though this observation has never been well studied and published, we wonder whether fetal anemia involves in fetal programming of adult cardiovascular disease and metabolic syndrome or not. This is due to the fact that alpha-thalassemia patients are usually anemic *in utero*, often more severe than in postnatal life, different from beta-thalassemia which is not associated with anemia *in utero*. We hypothesize that anemia *in utero* could involve in fetal programming of cardiovascular disease in adult life since the effect of anemic insults can theoretically impact on developing vital organs of the fetuses more serious than on the well-developed adult organs. The unhealthy heart of these fetuses can possibly be a predisposing factor of adult cardiovascular disease. This study is not aimed to directly link fetal anemia and adult diseases but we aimed to determine the effect of fetal anemia on the developing heart that might be a small piece of jigsaws to support the fetal programming.

Hemoglobin (Hb) Bart’s disease is a leading cause of fetal anemia in Asian and in particular Southeast Asian countries (1–4). The gene mutation (a deletion) frequency of Southeast Asia (SEA) gene is as high as 4.5–5%, resulting in a high prevalence of Hb Bart’s disease or the homozygous mutation (–SEA/–SEA) (5). The abnormality of red blood cells production due to Hb Bart’s disease causes severe fetal anemia which leads to hydrops fetalis (6). In these fetuses, the red blood cells have shortened life span and are excessively destroyed by reticuloendothelial (RE) system, causing fetal iron overload. Free iron due to heme destruction accumulates in the heart and may cause cardiac failure. Theoretically, such excessive free iron in cardiac tissue may cause myocardial tissue injury since non-transferrin bound iron (NTBI) is a toxic form of iron binding molecules. Moreover, NTBI can enter cells easier than transferrin and lead to cell apoptosis. Accordingly, we hypothesize that NTBI may play a role in pathogenesis of fetal myocardial damage leading to cardiac dysfunction and failure. The measurement of NTBI levels may be used to reflect iron overload. To date, the primary cause of cardiac failure in fetuses with Hb Bart’s disease is unclear, though fetal anemia can partly be responsible for an increase in cardiac output, finally resulting in cardiac failure. Nevertheless, hydropic fetalis caused by Hb Bart’s disease usually occurs in earlier gestation and more severe, unlikely to be completely explained by anemia.

In normal condition serum iron is bound to transferrin. Nevertheless, in cases of iron overload as seen in patients affected by homozygous beta thalassemia, serum transferrin is used up. As a result of transferrin depletion, iron binds to other kind of proteins, creating NTBI which is very responsive to redox reaction. Cell apoptosis and tissue injury can occur after transportation of NTBI into cells of many organs for example liver, spleen, brain, and heart. Iron overload can be assessed by measurement of serum NTBI levels. To the best of our knowledge, serum NTBI has never been measured in fetuses affected with alpha-thalassemia diseases. Labile Plasma Iron (LPI) represents a component of NTBI that is both redox active and chelatable, capable of permeating into organs and inducing tissue iron overload. LPI measurement can serve not only as indicators of impending iron overload but also as measures of the efficacy of iron chelation in eliminating a potentially toxic agent from plasma. LPI measures the iron-specific capacity of a given sample to produce reactive oxygen species (ROS) (7–10).

In fetal Hb Bart’s disease, the deterioration of fetal cardiac function usually begins in late gestation. Our team’s non-invasive study shows that fetal cardiac enlargement in response to anemia to increase cardiac output does not represent any sign of increased preload. The measurement of cardiac preload from inferior vena cava (IVC) or ductus venosus (3, 11) and evaluation of cardiac function by Tei index or shortening fraction are also in normal range although hydrops fetalis had already occurred. Biochemical markers for myocardial injury such as troponin-T not show any sign of cardiac failure (12). Such studies indicate that, apart from anemia, other mechanisms like iron overload may play a role and lead to cardiac failure in late state of the disease. To date, there is no strong evidence to conclude whether fetal cardiac failure is caused by volume overload or iron overload. We conducted this study to compare the levels of NTBI in fetuses affected with Hb Bart’s disease and those in unaffected fetuses and to determine the association between fetal cardiac function accessed by fetal echocardiography and the levels of NTBI in both groups.

## Patients and methods

A prospective cross-sectional study was conducted on pregnant women, attending the antenatal care clinic, Maharaj Nakorn Chiang Mai hospital, Chiang Mai University, between January 2017 and December 2019. The study was ethically approved by the institute review board. All participants were enrolled with written informed consent. The study population was pregnancies at risk of fetal Hb Bart’s disease who were scheduled to undergo prenatal diagnosis by cordocentesis at the. The inclusion criteria were as follows: (1) singleton pregnancies, (2) gestational age between 18 and 22 weeks, based on reliable last menstrual period and fetal biometry in the first half of pregnancies, and (3) pregnancies at risk of fetal Hb Bart’s disease, indicated by the carrier status of alpha-thalassemia-1 in both of the couples, based on PCR for alpha-thalassemia-1 (SEA type) gene. Patients whose have severe structural or chromosomal abnormality were excluded.

### Data and specimens collection

The recruited patients underwent cordocentesis performed by the MFM (maternal-fetal medicine) specialists. Preoperative preparation and the procedures were carried out as routine standard practice. The baseline characteristics were assessed and recorded at the time of recruitment, including age, gravidity, parity, gestational age, thalassemia screening result, complete blood count etc. Before cordocentesis, standard ultrasound were performed for fetal biometry to confirm gestational age and growth evaluation, anomaly screening and assessment of fetal cardiac function (Tei index and shortening fraction). Two-milliliters of fetal blood were obtained by cordocentesis for NTBI test (LPI kit), serum iron, transferrin, hemoglobin and hematocrit. After the procedure, the patients were taken standard of care. The diagnosis of fetal Hb Bart’s disease was made by Hb typing based on high-performance liquid chromatography (HPLC).

### Doppler ultrasound study for fetal cardiac function assessments

Fetal biometry and anomaly screening were firstly performed and cardiac function was evaluated. Average time of ultrasound examination was approximately 1 h per case. Sonographic evaluation was performed by the authors and research team (MFM specialists) after standardization of the sonographic techniques to minimize interobserver variations; using Voluson E8, or Voluson E10 (GE Medical Systems, Zipf, Austria) and Aloka alpha-10 scanners (Aloka Co., Ltd., Tokyo, Japan), equipped with a transabdominal curvilinear transducer of frequency 3.5–5 MHz.

On cardiac Doppler examination, Tei index was performed as follows (13): A cross-sectional image of the fetal thorax at the level of the four-chamber view (FCV) with an apical view of the heart was obtained. From the FCV, with slightly and cranially sweeping the ultrasound transducer, the origin of the aorta was visualized (five-chamber view). The Doppler volume sampling gate of 3 mm diameter was placed in the internal leaflet of the mitral valve (MV). In this location, owing to its proximity to the aortic valve (AV), the opening and closing AV clicks could be demonstrated. The angle of insonation was maintained as close as possible to 0^°^ and always less than 30^°^. The fastest velocity (15 cm/s) of the Doppler sweep was used to display Doppler tracings and the E/A waveform were displayed as positive flow. The Doppler gain was lowered and adjusted to clearly visualize the echoes representing the opening and closing clicks of the two valves at the beginning and at the end of the E/A (mitral valve) and aortic (AV) waveforms. A high-pass wall filter was used to avoid noises of wall motions or slow blood movements. The time cursor was placed at the beginning of each valve click. The three time periods were estimated as follows: (1) Isovolumetric contraction time (ICT) from the beginning of MV closure to AV opening; (2) Ejection time (ET) from atrioventricular valves open to closure; and (3) Isovolumetric Relaxation Time (IRT) from AV closure to MV opening. The Tei index is calculated as: (ICT + IRT)/ET. The mean of the best three consecutive recordings was considered as representative for each fetus.

Ventricular shortening fraction (SF) measurement (14): On real-time 2-dimension ultrasound examination, the standard four-chamber view was firstly identified and set to the center of screen and then adjusted to get the interventricular septum (IVS) to align horizontally as much as possible. Secondly, M-mode was activated and the M-line was set to be perpendicular to the IVS at the level of greatest dimension of the ventricles, usually near the atrioventricular valves. The measurements were made on the M-mode tracings at the end-diastole and the end-systole by placing the M-mode cursor at the level of the greatest dimension. The dimensions were measured, including LVID (left ventricular internal dimension) which was measured from the endocardium of the left ventricular wall to the endocardium of the left side of the IVS; and RVID (right ventricular internal dimension) which was measured from the endocardium of the right ventricular wall to the endocardium of the right side of the IVS. These parameters were measured in the same tracings at both end-diastole and end-systole. The best measurements were selected and recorded for analysis. Left ventricle shortening fraction (LVSF) and right ventricle shortening fraction (RVSF) were calculated using equation: SF = Dimension at end-diastole—Dimension at end-systole/Dimension at end diastole.

### Measurement of plasma non-transferrin bound iron concentration by Labile Plasma Iron

The FeROS^®^ LPI kit was a fluorescence e-based assay intended for the *in vitro* qualitative detection of the redox active LPI fraction of NTBI (7). The serum or heparinized plasma that was freshly collected on the day of the test or immediately frozen at −20 to −70^°^C (up to 2 h after collection). Samples could be stored at −20^°^C for a maximum of 3 months and at −70^°^C or below up to 6 months, provided samples were not thawed more than twice. No citrate or EDTA tubes were used. Blood samples were advised to be withdrawn at trough levels of plasma chelator (drugs used to remove extra iron from the body, including natural compounds derived from microorganisms such as siderophores and synthetic iron chelators), just before the 1st daily intake of deferasirox or deferiprone or just before infusion of deferioxamine, as LPI levels could be affected by the presence of iron chelator in plasma.

### Statistical analysis

Statistical techniques were performed using the statistical package for the social sciences (SPSS) software version 26.0 (IBM Corp., Released 2019. IBM SPSS Statistics for Windows, Version 26.0, IBM Corp., Armonk, NY, USA). Quantitative analyses were expressed as mean + standard deviation (SD) or median (interquartile range: IQR), as appropriate. Student’s *t*-test or Mann-Whitney Test were performed as appropriate to compare the continuous variables and Chi-square test to compare the categorical variables. The statistically significant value was accepted with *P* < 0.05.

## Results

A total of 56 pregnant women at risk of fetal Hb Bart’s disease were recruited to the study. Of them, 50 women, consisting of 20 fetuses with Hb Bart’s disease and 30 unaffected fetuses, underwent complete fetal parameter measurement and cardiac examinations and were available for analysis. All of the affected cases presented with pre-hydropic features. All maternal baseline characteristics of both groups were not significantly different (*p*-value > 0.05), as presented in Table 1. However, some fetal baseline parameters were different significantly (*p*-value > 0.05). Note that nearly all of fetuses affected with Hb Bart’s disease had a lower level of hemoglobin and hematocrit than that of the unaffected group. Moreover the white blood cell and nucleated red blood cell levels were also significantly lower when compared with those of the unaffected group. Mean platelet volume (MPV) levels were significantly higher in the affected fetuses (*p*-value < 0.001). There was a significant increase in the level of serum iron in the affected group (median: 22.7 vs. 9.7; *p*-value: 0.013) and also a significant increase in non-transferrin binding iron (NTBI) when compared with those of the unaffected fetuses (median 0.11 vs. 0.07; *p*-value 0.046), as presented in Table 1.

**TABLE 1:** Baseline characteristics of both groups.

	Hb Bart’s group *N* = 20	Non-Hb Bart’s group *N* = 30	*P*-value
Age	28.1 ± 7.1	29.2 ± 5.4	0.518
Parity: nulliparous/parous	14/6	20/10	0.804
Gestational age	19. 5 ± 2.4	19.1 ± 1.5	0.572
Maternal blood serum			
Hct	35.2 ± 3.1	33.5 ± 3.0	0.064
MCV	66.3 ± 3.0	67.1 ± 7.6	0.640
Fetal blood test			
Serum Iron	22.7 (37.3)	9.7 (14.0)	0.013
Serum LPI	0.11 (0.11)	0. 07 (0.12)	0.046
Hb	6.0 ± 1.6	10.2 ± 2.2	< 0.001*
Hct	27.8 ± 6.9	31.7 ± 6.0	0.044*
WBC	24.236 ± 2.121	10.608 ± 2.794	0.070
NRBC	2.217 ± 1.465	350 ± 1.385	< 0.001*
Corrected WBC	1.305 ± 1.468	2.717 ± 1.770	0.005*
Plt	193.600 ± 102.770	167.400 ± 92.767	0.353
MPV	12.1 ± 1.1	10.5 ± 1.1	< 0.001*
RET	18.9 ± 3.2	19.1 ± 3.8	0.850
IPF	10.5 ± 7.4	8.5 ± 9.4	0.431

Hb, hemoglobin; Hct, hematocrit; IPF, immature platelet fraction; LPI: labile plasma iron; MCV, mean corpuscular volume; MPV, mean platelet volume; NRBC, nucleated red blood cell; Plt, platelets; RET, reticulocyte count; WBC, white blood cell count. *Statistically significant.

The ultrasound findings in both groups were significantly different in a part of pre-hydropic signs; including fetal cardiac dimensions (cardio-thoracic ratio, CTR) (*p* < 0.001), liver length (*p* < 0.001), placental thickness (*p* < 0.001) and middle cerebral artery peak systolic velocity (MCA-PSV) (*p* < 0.001). Nevertheless, there was no significant difference in the results of umbilical artery study, as presented in Table 2.

**TABLE 2:** Prenatal 2D-ultrasound findings and spectral Doppler studies.

	Hb Bart’s group *N* = 20; median (IQR) or mean ± *SD*	Non-Hb Bart’s group *N* = 30; median (IQR) or mean ± *SD*	*P*-value
CTR	60.7 ± 6.5	45.3 ± 3.2	< 0.001*
Liver length (cm)	2.9 ± 0.7	2.1 ± 0.4	< 0.001*
Placental thickness (cm)	3.1 ± 0.7	2.1350	< 0.001*
MCA-PSV (cm/sec)	40.6 ± 8.6	24.1 ± 7.6	< 0.001*
MCA-PSV (MoM)	1.6 ± 0.3	1.0 ± 0.3	< 0.001*
UAS/D	4.5 (1.2)	3.7 (1.2)	0.055
UA PI	1.44 (0.35)	1.24 (0.28)	0.056
UA RI	0.78 (0.14)	0.73 (0.09)	0.081

CTR, cardiac: thoracic diameter ratio; IQR, interquartile range; MCA-PSV, middle cerebral artery—peak systolic velocity; MoM, multiple of median; SD, standard deviation; UA PI, umbilical artery pulsatility index; UA RI, umbilical artery resistant index; UA S/D, umbilical artery systolic/diastolic velocity ratio. *Statistically significant.

In comparison of fetal cardiac function parameters between those in the affected and the unaffected group, myocardial performance (Tei) index in both sides of the heart and isovolumetric contraction time (ICT) were significantly increased in the affected group; (left Tei: *p* < 0.001, Right Tei: *p* = 0.008; left ICT: *p* < 0.001, right ICT: *p* < 0.001, respectively). However, other parameters of the fetal cardiac function were not significantly different between both groups, as presented in Table 3.

**TABLE 3:** Comparisons of fetal cardiac function of both groups.

	Hb Bart’s group *N* = 20; median (IQR) or mean ± *SD*	Non-Hb Bart’s group *N* = 30 median (IQR) or mean ± *SD*	*P*-value
**Left heart**			
● Tei index	0.57 (0.15)	0.46 (0.10)	< 0.001*
● ICT	50.0 (14)	36.0 (13)	< 0.001*
● IRT	42.5 (9.0)	40 (9.0)	0.064
● ET	165 (27)	166 (19)	0.851
**Right heart**			
● Tei index	0.55 (0.17)	0.46 (0.10)	0.008*
● ICT	44.0 (12.0)	32.0 (13.3)	< 0.001*
● IRT	47.0 (11.0)	42.0 (9.8)	0.155
● ET	165 (24)	164 (20)	0.699
Left cardiac output	316 ± 129	275 ± 89	0.186
Right cardiac output	443 ± 159	343 ± 136	0.022
E/A ratio of mitral valve	0.59 (0.14)	0.56 (0.12)	0.316
E/A ratio of tricuspid valve	0.67 (0.15)	0.63 (0.11)	0.162
LV shortening fraction	32.3 (19.4)	37.6 (9.3)	0.332
RV shortening fraction	36.6 (12.1)	41.5 (15.3)	0.175
DVa	23.5 (28.0)	16.0 (15.4)	0.216

DVa, a-wave of the ductus venosus Doppler waveform; E/A, E-wave/A-wave ratio; ET, ejection time; ICT, Isovolumetric contraction time; IQR, interquartile range; IRT, isovolumetric relaxation time; LV, left ventricle; RV, right ventricle; SD, standard deviation. *Statistically significant.

**FIGURE 1:**
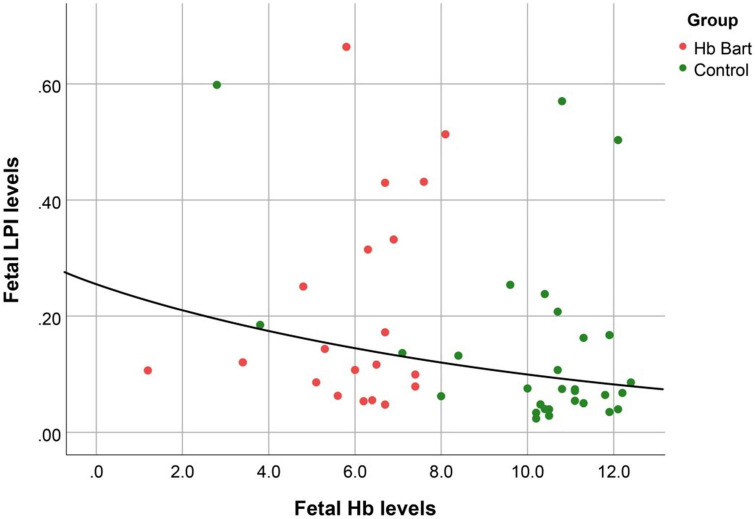
The correlation between fetal LPI (Labile Plasma Iron) levels and fetal hemoglobin levels (Exponential function; *R*^2^: 0.095; *p*: 0.030).

**FIGURE 2:**
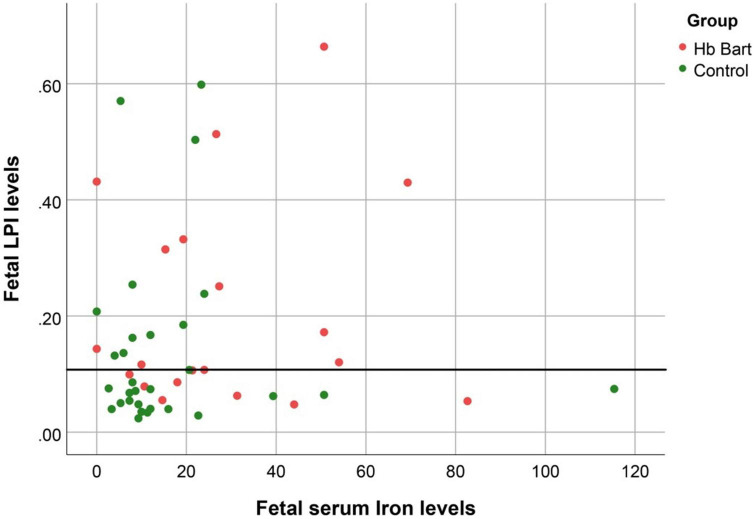
The correlation between fetal LPI (Labile Plasma Iron) levels and fetal serum iron levels (S function; *R*^2^: 0.045; *p*: 0.138).

**FIGURE 3:**
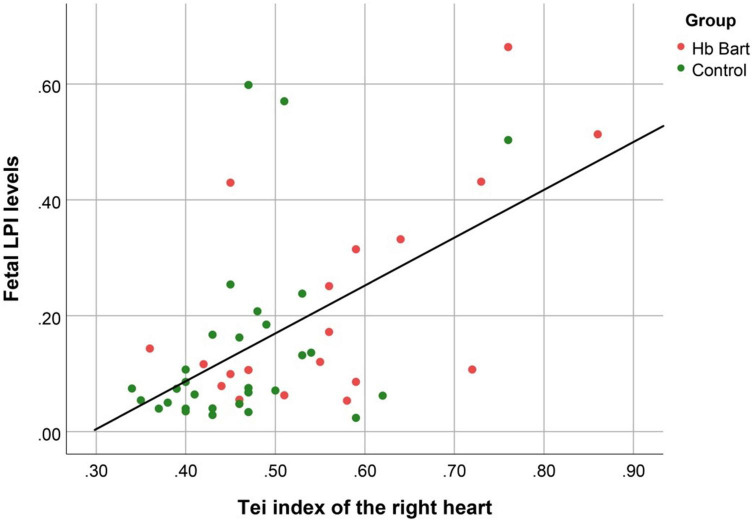
The correlation between fetal LPI (Labile Plasma Iron) levels and fetal right myocardial performance index (Tei) (Linear function; *R*^2^: 0.326; *p*: < 0.001).

**FIGURE 4:**
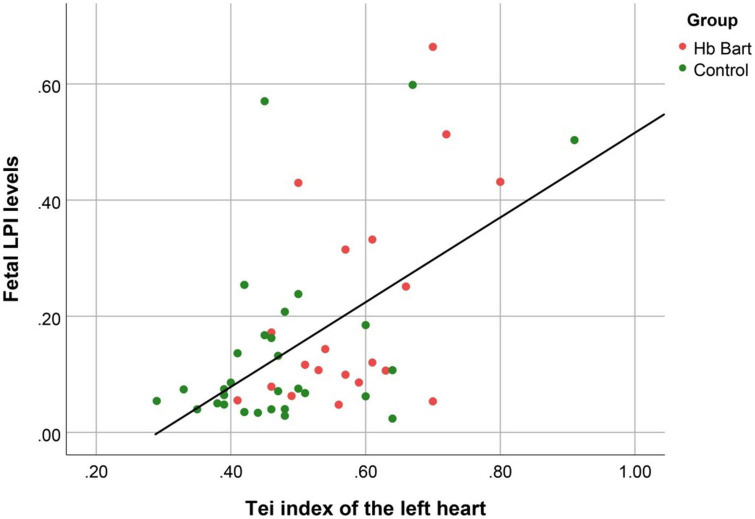
The correlation between fetal LPI (Labile Plasma Iron) levels and fetal left myocardial performance index (Tei) (Linear function; *R*^2^: 0.291; *p* < 0.001).

**FIGURE 5:**
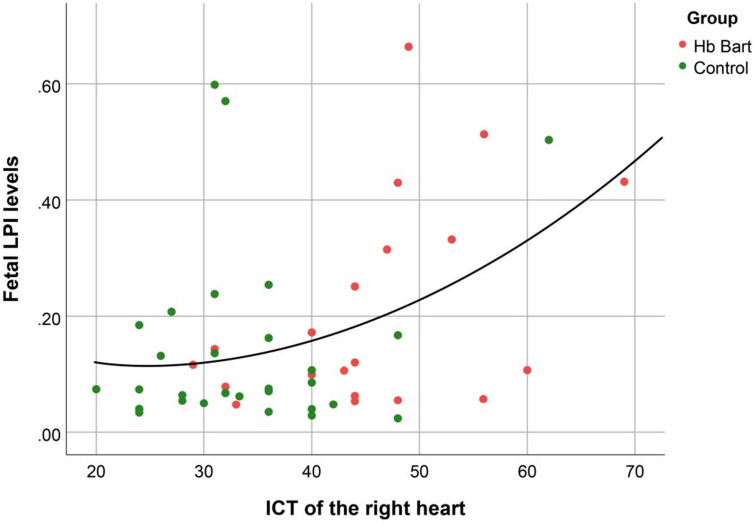
The correlation between fetal LPI (Labile Plasma Iron) levels and fetal right isovolumetric contraction times (ICT) (Quadratic function; *R*^2^: 0.170; *p*: 0.013).

**FIGURE 6:**
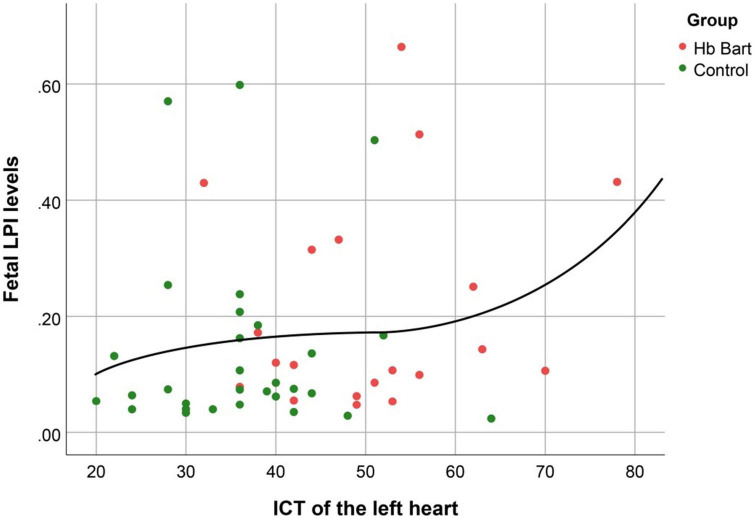
The correlation between fetal LPI (Labile Plasma Iron) levels and fetal left isovolumetric contraction times (ICT) (Cubic function; *R*^2^: 0.044; *p*: 0.554).

Fetal LPI levels were significantly correlated inversely with fetal hemoglobin levels (*p*-value: 0.030) but not significantly correlated with the fetal serum iron levels (*p*-value: 0.138), as shown in Figures 1, 2. Fetal LPI levels were significantly correlated positively with myocardial performance index (Tei) of both sides (right Tei index: *R*^2^: 0.326; *p*: < 0.001), (left Tei index: *R*^2^: 0.291; *p* < 0.001), as shown in Figures 3, 4. Additionally, fetal LPI levels were also significantly correlated positively with ICT of the right side (*R*^2^: 0.170; *p*: 0.013), but not significantly correlated with left ICT (*R*^2^: 0.044; *p*: 0.554). as presented in Figures 5, 6. Multivariate analysis for correlation of fetal LPI and cardiac function was performed to adjust for possible confounders, including maternal age, gestational age, hemoglobin; mean corpuscular volume; reticulocyte count; nucleated red blood cell; white blood cell count; platelet count; mean platelet volume; and immature platelet fraction. On multivariate analysis, fetal LPI levels were independent factors on cardiac dysfunction; significantly associated with Tei index of both sides, and ICT of the right side as presented in Table 4.

**TABLE 4:** Correlation between fetal LPI (Labile Plasma Iron) levels and cardiac function based on univariate analysis and multivariate analysis (adjusted for maternal age, gestational age, hemoglobin; mean corpuscular volume; reticulocyte count; nucleated red blood cell; white blood cell count; platelet count; mean platelet volume; and immature platelet fraction).

Parameters of cardiac function as a function of LPI levels	Univariate analysis		Multivariate analysis	
	Standardized coefficients beta	*P*-value	Standardized coefficients beta	*P*-value
**Left heart**				
● Tei index	0.543	< 0.001	0.417	0.007
● ICT	0.185	0.198	0.092	0.577
● IRT	0.387	0.006	0.285	0.054
● ET	–0.371	0.008	–0.194	0.296
**Right heart**				
● Tei index	0.575	< 0.001	0.496	0.002
● ICT	0.388	0.005	0.448	0.008
● IRT	0.465	0.001	0.274	0.052
● ET	–0.271	0.057	–0.012	0.948
Left cardiac output	0.063	0.665	–0.040	0.814
Right cardiac output	–0.063	0.663	–0.121	0.439
E/A ratio of mitral valve	–0.066	0.650	–0.041	0.807
E/A ratio of tricuspid valve	0.038	0.793	–0.027	0.888
LV shortening fraction	0.116	0.423	0.008	0.966
RV shortening fraction	–0.144	0.318	–0.031	0.867
DVa	0.231	0.106	0.182	0.283

DVa, a-wave of the ductus venosus Doppler waveform; E/A, E-wave/A-wave ratio; ET, ejection time; ICT, Isovolumetric contraction time; IQR, interquartile range; IRT, isovolumetric relaxation time; LV, left ventricle; RV, right ventricle; SD, standard deviation.

## Discussion

This study contributes to our understandings on fetal response to anemia in pre-hydropic phase, using Hb Bart’s disease as a study model, as follows: (1) LPI or NBTI levels increased significantly in the affected fetuses, indicating fetal long exposure to oxidative stress. (2) LPI levels were significantly correlated with cardiac dysfunction, reflected by increased Tei index and ICT and volume overload, reflected by increased cardiac output; (3) Prolonged ICT representing systolic dysfunction develops earlier than IRT which represent diastolic dysfunction; (4) Cardiac dysfunction firstly appears on the right side; (5) Afterload represented by umbilical artery Doppler studies is not significantly increased in spite of a significant increase in cardiac output; and (6) Other findings are confirmatory to existing knowledge such as cardiomegaly and increased MCA-PSV in fetal anemia, etc.

This study does not provide evidence to link fetal anemia to cardiovascular disorders in adult life. However, we provide evidence that intrauterine anemia can cause cardiac dysfunction, firstly prolonged ICT and increased Tei index, either *via* an increase in LPI, longstanding volume load or direct effect of anemic hypoxia on myocardial cells. Since increased LPI or NTBI is associated with oxidative stress that can cause mitochondrial dysfunction or myocardial cell damage leading to compromised heart, intrauterine longstanding anemia due to non-lethal anemia, especially HbH disease and variants may predispose to adult cardiovascular disease. Several previous studies indicate that fetal growth restriction which can compromise heart because of hypoxia, can be an epigenetic cause of cardiovascular disorders in adult life. Accordingly, our study, which linked fetal anemia to an increase in LPI levels, cardiac dysfunction and overworked heart, suggests that fetal anemia may hypothetically increase risk of adult cardiovascular diseases in future life.

We believe that anemia in adult but not *in utero* like beta-thalassemia or sickle cell disease may not suffer from abnormal fetal programming of cardiovascular disease like alpha-thalassemia such as Hb H disease and variants. Convincingly, newborns of alpha-thalassemia have more compromised heart than those of beta-thalassemia because anemia due to alpha-thalassemia occurs during fetal life, the critical time for developing vital organs like the heart brain and liver. However, cardiovascular diseases among these group have not been explored.

### Non-transferrin binding iron, serum iron and hemoglobin levels

This study showed that Hb levels were significantly decreased in the affected fetuses and also showed an increase in serum iron and non-transferrin binding iron (NTBI) levels when compared with those in the controls. The increase in NTBI levels was strongly correlated with a decrease in fetal Hb levels, indicating that the anemia was a result of hemolysis, reflected by increased NTBI levels.

### Fetal cardiac function and non-transferrin binding iron levels

Iron overload can stimulate reactive oxygen species (ROS) production by Haber-Weiss and Fenton reactions (15, 16), in which hydroxyl and hydroxide ions are generated from the reaction of H2O2 and superoxide ion catalyzed by iron. Destruction of cellular lipids, proteins, and DNA is caused by rising of ROS levels. Furthermore, disruption of organelles such as lysosomes and mitochondria can occur in the environment of increased ROS (15, 16). Previous studies have demonstrated that mitochondrial dysfunction accounts for cardiac failure in chronic iron overload (17, 18).

In adults with chronic iron overload or beta-thalassemia major, ROS is accumulated in cytoplasmic labile iron pool. Those irons are available for Fenton-type reactions, part of cellular respiration, leading to the conversion of Fe2 + to Fe3 + which generate hydroxyl radical (free radical). In normal cell status, these hydroxyl free radicals are inactivated by scavenger enzyme (glutathione peroxidase and catalase). On the other hand, in condition with high iron level, excessive free radical leads to cellular damage by increasing peroxidation, in turn causing destruction of protein, membrane lipid or DNA. The deterioration of lipid and increasing peroxidation precipitate elevation of cytotoxic agents such as malondialdehyde (MDA) (19, 20). This process is recognized in homozygous beta thalassemia disease or primary hemochromatosis with cardiomyopathy. This product can combine with protein (aldehyde-protein adducts) give rise to cellular dysfunction due to loss of protein function (21).

It is noteworthy that in fetal anemia the heart is overworked, indicated by increased Tei index, which already occurs before development of hydrops fetalis. The finding is consistent with that reported by Luewan et al. (11), who demonstrated that in the first half of pregnancy, Tei index in fetal Hb Bart’s disease was significantly higher than that in normal fetuses.

We found an inversed correlation between worsening cardiac function (increased Tei index) and an increase in NTBI levels. Note that ICT value of the right heart was also significantly increased with NTBI levels, whereas such a correlation was not significant for the left heart, signifying that fetal anemia effects firstly on the right heart, though we believe that an increase in NTBI levels have the same impact on both the left heart and right heart myocardial cells. This might be explained by the fact that volume load of the right side is normally greater than that of the left side, though the effect of volume load on cardiac dysfunction is subtle in early phase, leading to cardiac dysfunction is more pronounced in the right side. The mechanism that higher NTBI levels negatively impact on cardiac function is unclear. Nevertheless, it is known that the excessive amount of iron increases oxidative stress and inflammation process which can affect the functioning of the cells, especially cells that need a lot of energy, such as the heart and brain. In heart failure or late stage of cardiac dysfunction, Tei index is increased together with prolonged ICT and IRT, reflecting both systolic and diastolic dysfunction, respectively (22, 23). Accordingly, an increase in Tei index and ICT but not IRT in this study suggests that fetal anemia in early phase or pre-hydropic phase firstly affect the systolic function. However, it is possible that diastolic dysfunction appears at the same stage of fetal anemia if assessed by other cardiac function analysis technologies. When the anemic insults become more pronounced, both ICT and IRT are expected to be prolonged. Notably, an increase in LPI was significantly correlated with increased Tei index and increased ICT but it was not significantly correlated with volume load represented by increased cardiac output. The finding indicated that cardiac dysfunction responsible for subsequent hydrops fetalis secondary to anemia might, in part, directly associated with an increase in NTBI levels, independently from the mechanism induced by volume overload.

Additionally, we also observed that whereas Tei index was significantly increased and ICT was significantly prolonged in affected fetuses, afterload reflected by umbilical artery Doppler waveforms were still preserved. Interestingly, in spite of volume overload in cases of fetal anemia the afterload was not significantly increased. Our findings might be explained by previous studies on fetal lambs (24, 25), which have demonstrated that the oxygen-carrying capacity is reduced because of anemia, leading to a compensatory reduction in systemic vascular resistance or afterload and increase in cardiac output.

The limitations of this study are as follows: (1) This study is preliminary and there is no long-term data to support hypothesis of possible predisposing to cardiovascular disease in adult life. (2) The sample size is relatively small. (3) The natural course of anemia secondary to Hb Bart’s disease which we used as a study model could not perfectly represent anemia caused by other disorders. Additionally, it should be emphasized that early detection of cardiac dysfunction in this study is mainly based on conventional spectral Doppler (Tei index). Though it has advantages of assessment for both systolic and diastolic dysfunction with acceptable reproducibility and widely use in practice, it has some limitations including less reliability when applied to the right side in late gestation, variability among reference values in the literature, need of proper presets established by the echocardiographer for each system because of variations among different machine platforms, and influence of scanning speed and filter. This may not be the best tool compared to other new technology. However, several novel techniques for assessment of cardiac function, such as speckle tracking technology or fetal HQ, or automated measurements on high-end ultrasound equipment need further validation with more large-scale studies for its reproducibility, and its comparability to existing cardiac function tools before incorporating into clinical practice.

### Gap of knowledge

This study provides evidence that fetal anemic hypoxia can compromise the developing heart *via* oxidative stress or overloaded dysfunction. However, whether the compromised heart *in utero* can cause adult cardiovascular diseases or not is yet to be elucidated. We suggest that cardiovascular diseases and metabolic syndrome among adults who experience anemia *in utero* like alpha-thalassemia disease, especially Hb H disease and variants should be explored or compared with normal controls.

## Conclusion

New insights concerning fetal response to Hb Bart’s disease include: (1) LPI or NTBI levels significantly increased, and their levels are significantly correlated with cardiac dysfunction; (2) Prolonged ICT develops earlier than IRT; (3) Afterload is not significantly increased in spite of a significant increase in cardiac output; and (4) Cardiac dysfunction firstly appears on the right side. However, this is a preliminary study on NTBI in fetuses affected by Hb Bart’s disease. Accordingly, further studies including long-term data are required to support a role of fetal anemia as well as increased fetal serum NTBI levels in development of subsequent heart failure or cardiac compromise among the survivors, possibly predisposing to cardiovascular disease in adult life.

## Data availability statement

The raw data supporting the conclusions of this article will be made available by the authors, without undue reservation.

## Ethics statement

The studies involving human participants were reviewed and approved by the Faculty of Medicine, Chiang Mai University (Ethics Committee 4). The patients/participants provided their written informed consent to participate in this study.

## Author contributions

PJ and TT conceived and designed the study. PJ performed the clinical experiment and wrote the manuscript. SK, RS, TJ, and NC performed laboratory experiment. TT analyzed the data. TT and NC edited the manuscript. All authors contributed to the article and approved the submitted version.
